# A theoretical and experimental model of flow characteristics in subretinal injections

**DOI:** 10.1371/journal.pone.0344836

**Published:** 2026-03-20

**Authors:** Reza Ladha, Benjamin Merveille, Roland Wyns, Benoit Scheid, François Willermain, Marc D. de Smet

**Affiliations:** 1 Departments of Ophthalmology, CHU Saint-Pierre and CHU Brugmann, Brussels, Belgium; 2 Université Libre de Bruxelles, Brussels, Belgium; 3 Biomedical Department, CHU Saint-Pierre, Brussels, Belgium; 4 TIPs-μfluidics, Université Libre de Bruxelles, Brussels, Belgium; 5 Department of Ophthalmology, Leiden University, Leiden, The Netherlands; 6 New York Eye and Ear Institute of Mt Sinai, Icahn School of Medicine, New York, United States of America; 7 MIOS sa, Lausanne, Switzerland; Mayo Clinic Minnesota, UNITED STATES OF AMERICA

## Abstract

Subretinal injections (SI) are used to deliver gene therapies for inherited retinal diseases, yet the optimal injection parameters remain undefined. This study used theoretical and experimental models to quantify the relationship between injection pressure, flow dynamics, and residual flow. A theoretical model (TM) was developed based on the Hagen-Poiseuille law and the theory of a jet immersed in the same liquid. An experimental model (EM) was constructed to allow for measuring flow and residual flow across injection pressures ranging from 0 to 20 psi. We assessed the effects of ambient pressure, injection system tubing length, and syringe priming technique. A minimum pressure of 6 psi was required to generate a detectable flow in the EM. Jet speed increased with the square root of injection pressure, aligning with theoretical predictions. Residual flow persisted for 28–47 seconds after injection and increased logarithmically with injection pressure. Elevated ambient pressure (45 mmHg) only reduced flow at lower injection pressures. The “lock-and-load” priming method decreased jet speed and increased residual flow compared to the “load-and-lock” method. Both TM and EM quantified SI flow dynamics, with EM demonstrating residual flow at all tested pressures. To minimize complications, clinicians should use the lowest injection pressure and allow sufficient time for the cannula to be withdrawn from the subretinal space.

## Introduction

There are three methods for gene delivery in inherited retinal diseases: intravitreal, suprachoroidal, and subretinal injection (SI). The subretinal approach is the most precise but also the most surgically demanding. Several protocols have been proposed for SI [1–3]. They all emphasize the need for appropriate training, acknowledge the steep learning curve, and stress the importance of managing crucial surgical steps optimally.

There are many challenges associated with delivering gene vectors [4]. Complications such as chorioretinal atrophy are increasingly being reported not only at the injection site, but around their edges [5–7]. Several potential causes have been suggested, including the direct toxic effects of the vector, abnormal vectors, immunological responses, and surgical technique [8–10]. Of the surgical factors identified, excessive flow during subretinal injection has been suggested to be a potential cause of mechanical damage to photoreceptors and retinal pigmented epithelial (RPE) cells [11,12].

Reflux during the procedure is another major concern. In a previous study, we demonstrated that removing the cannula from the subretinal space caused fluid to escape not only from the retinal incision site, but also from the cannula tip [13]. This can possibly lead to adverse immune responses [14–18].

The precise relationship between the injection pressure and resulting flow rates, as well as the persistence of a residual flow after injection cessation, has not been studied in the current patient protocols. Rossi et al. employed an empirical approach to recover the linear relationship between flow rate and pressure drop associated with frictional head loss [19]. However, when accounting for the additional pressure drop associated with head loss at the exit inside the intraocular cavity, the correlation becomes quadratic.

We used a theoretical model (TM) for laminar flow to quantify the relationship between injection pressure and jet speed. Then, we developed a physical experimental model (EM) to test the limits of the TM and quantify unintended residual flow persistence after injection. Additionally, our models enabled us to evaluate various injection protocols under different simulated conditions.

## Methods

### Theoretical modelling of subretinal injections

We created a theoretical model (TM) of an injection system (SI) by modelling the following components: a vitrectomy machine (BL15455 Stellaris ELITE, Bausch and Lomb, Vaughan, Canada), viscous fluid injection (VFI) tubing (BL7600 Universal Viscous Fluid Control Pack, Bausch and Lomb, Vaughan, Canada), a 1mL syringe (Model 3275 Microdose Injector, MedOne Surgical Inc., Sarasota, FL, USA), a 5 cm short extension tube (Model 3223 Extension Tube, MedOne Surgical Inc., Sarasota, FL, USA), and distally, a subretinal injection cannula with a 41 g inner diameter polyamide tip of 5 mm in length (Model 3219 Polytip Cannula,MedOne Surgical Inc., Sarasota, FL, USA).

To create the theoretical model (TM), the internal diameter D and length L of each component were obtained either from either the manufacturer’s specifications or direct measurements of the product itself.

Table 1 summarizes this information.

**Table 1:** The hydrodynamic resistance (*R*_*hyd*_), internal diameter (D), and length (L) of each part of the injection system of interest.

	*D* ´10^−3^ [m]	*L* ´10^−3^ [m]	*R*_*hyd*_ ´10^12^ [Pa.s/m^3^]
**Distal Polyamide section of Model 3219 Polytip Cannula MedOne Surgical Inc., Sarasota, FL, USA)**	0.071	5	8.02
**Distal Polyamide section of Model 3255 Polytip Cannula MedOne Surgical Inc., Sarasota, FL, USA)**	0.071	2	3.21
**Proximal needle section of Model 3219/3255 Polytip Cannula MedOne Surgical Inc., Sarasota, FL, USA)**	0.26	35	0.31
**5 cm short extension tube (Model 3223 Extension Tube MedOne Surgical Inc., Sarasota, FL, USA)**	0.5	150	0.10

### Modelling of the injection system

The following formula was used to calculate the hydrodynamic resistance in the injection system:


$$R_{hyd}=\frac{\text{1}\text{2}\text{8}\mu L}{\pi D^{\text{4}}}$$ (1)

where μ is the dynamic viscosity of the liquid. Water with μ=0.001 Pa· s was used for the analysis.

We used the Hagen-Poiseuille law to quantify the flow rate Q of the liquid exiting the injection system [20],


$$Q=\frac{\Delta P-P_{am}}{R_{hyd,eff}}$$ (2)

where ΔP is the pressure imposed on the liquid side of the piston inside the syringe; *P*_am_ the ambient pressure in the vitreous cavity; $R_{hyd,eff}$ the effective hydrodynamic resistance, which corresponds to the sum of the hydrodynamic resistances of the tubing components in series. All pressures were measured relative to atmospheric pressure.


$$\Delta P=P-P_{min}$$ (3)

where *P* is the injection pressure, and *P*_min_ is the minimum air pressure necessary to overcome the static friction force between the piston and cylinder.

The mean velocity of the liquid within the injection system corresponds to the internal diameter of the tubing at any given location and the flow,


$$v=\frac{\text{4}Q}{\pi D^{\text{2}}}$$ (4)

Based on these equations (1–4), we can quantify the flow rate Q and mean velocity v of the liquid exiting the injection setup as a function of the selected injection pressure on the vitrectomy machine. We can also analyze the impact of the cannula’s diameter and length, as well as the changes in the viscosity of the injected solution.

As for the immersed liquid jet exiting the cannula, the jet speed ($v_{jet}$) is defined as the distance traveled by the jet ($z_{jet}$) during the injection time ($t_{inj}$). From the Landau jet theory [21], one can derive from momentum conservation that the speed of a round jet decreases inversely with the distance traveled by the jet. More specifically, for a non-punctual source, the following correlation can be used [22]


$$v_{jet}=K\frac{v\;D}{z_{\text{jet}}}\;$$ (5)

This is valid for $z_{\text{jet}}\gg D$, where K is a constant that will be fitted with the experimental data. This constant accounts for the non-ideal effects such as the confinement between the two parallel plates in the experimental set-up and the effect of gravity. Since by definition, $v_{jet}=z_{jet}/t_{inj}$, one can rewrite (5) as follows:


$$v_{jet}=\sqrt{\frac{K\;v\;D}{t_{\text{inj}}}}\;$$ (6)

This shows that, for a fixed injection time, the jet speed varies with the square root of the mean velocity, or, equivalently, the injection pressure. This is because equations (4) and (2) show that the mean velocity varies linearly with the injection pressure.

### The experimental model and its components

**Fig 1:**
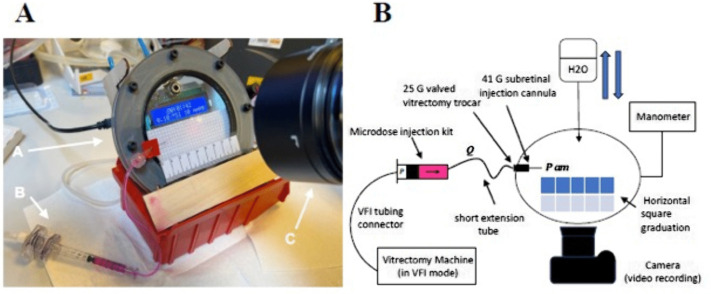
Description of the experimental model. A/ The injection chamber **(A)** has clear polycarbonate walls and is equipped with a manometer and a digital display to monitor injection parameters (injection pressure, pressure within the chamber, and injection timing), which are selected on a vitrectomy machine. The chamber has a sealed vitrectomy trocar opening that allows the injection system to enter **(B)**. The injection system consists of a subretinal injection cannula that is connected to a vitrectomy machine set to viscous fluid injection mode and filled with ctHB solution. A video camera **(C)** is optimally positioned in front of the chamber to record the injection, and the parameters are displayed on the digital monitor. This completes the experimental setup. B/ Schematic representation of the injection system and its relationship with the experimental model, which is positioned in front of a video camera.

An experimental model (EM) was created to confirm the validity range of the TM and study fluid extrusion characteristics when entering the subretinal space. Experiments were conducted at room temperature. We used the same injection system components as those on which we based our TM. Fig 1A shows an artificial, fluid-filled injection chamber in which colored fluid egressing from the cannula tip could be observed. The ambient pressure within the chamber was measured in real time and found to vary. The simulated SI was recorded using a video camera inside the chamber (Fig 1).

### Injection system

The injection system consisted of the following components: a vitrectomy machine (BL15455 Stellaris ELITE, Bausch and Lomb, Vaughan, Canada), a viscous fluid injection (VFI) tubing (BL7600 Universal Viscous Fluid Control Pack, Bausch and Lomb, Vaughan, Canada), a 1mL syringe (Model 3275 Microdose Injector, MedOne Surgical Inc., Sarasota, FL, USA), a 5 cm short extension tube (Model 3223 Extension Tube, MedOne Surgical Inc., Sarasota, FL, USA), and a subretinal injection cannula with a 41g inner diameter polyamide tip of 5 mm in length (Model 3219 Polytip Cannula,MedOne Surgical Inc., Sarasota, FL, USA). The silicone oil injection mode was selected on the vitrectomy machine interface. The syringe was then filled with ctHB calibration solution (Radiometer America Inc., Brea, CA, USA). This colored solution, which has a viscosity comparable to water, enables optimal visualization within the injection chamber.

### Injection chamber

The injection chamber consisted of two transparent external circular sheets of polycarbonate (15 cm in diameter) that were attached to a 4 mm circular spacer of polyactic acid (PLA) along their peripheral surfaces over 360°. This formed an inner, closed chamber with a volume of 84 cm^3^.

An opening was made in the anterior surface of the model, in which a valved vitrectomy trocar (BL5282 25G ESA Kit-valved, Bausch and Lomb, Vaughan, Canada) was permanently sealed. This enabled a 10° oblique entry of the injection cannula into the injection chamber. The inferior inner surface was lined with a 5 mm square graduation.

Tow opening on the posterior surface of the injection chamber allowed for external connection to a water bottle and a manometer. By adjusting the height of the water bottle, the pressure in the chamber could be altered and its effect on flow out of the cannula could be observed.

We used a manometer (IDA-5; Fluke Biomedical, Washington, USA) to continuously record the ambient pressure within the chamber. The manometer, the chamber, and the external connections were securely attached to a specially designed balsa support, along with a digital display.

### Video recording

We used a video camera, an Olympus OM-D-E-M1X (OM Digital Solutions Corporation, Tokyo, Japan), to complete the setup. The camera was positioned in front of the injection chamber, as shown in Fig 1. It recorded the flow of each injection and the information visible on the digital display, including the injection pressure selected on the vitrectomy machine, the ambient pressure within the chamber, and the timing of the injection. After the experiments, the video recordings were visually analyzed using the OM Workspace software (OM Digital Solutions Corporation, Tokyo, Japan).

### Experimental protocol

Experiments were conducted at room temperature. Prior to the injection, the ambient pressure within the injection chamber was set to 15 or 45 mmHg. Then, the solution was loaded into the injection syringe using a separate, pre-filled syringe according to the so-called “load-and-lock” method. Once an adequate amount, free of visible bubbles, was present in the barrel of the injection syringe, the cannula was connected to its tip, and the injection system was purged. Alternatively, the injection syringe was primed using the “lock-and-load” methodology. To this end, the injection system was pre-assembled, and the syringe was filled by drawing up the injection fluid through the injection cannula without removing the air trapped in the syringe between the plunger and the solution. Additionally, we compared VFI tubing of standard length with tubing reduced to three-quarters of its normal length.

Prior to the injection, the operator determined the injection pressure of the vitrectomy machine (set on silicone injection mode). The foot pedal of the vitrectomy machine was pressed for 5 s to activate the viscous fluid injection function. The time at which injection began and stopped was recorded using a timer. The fluid ejected from the cannula tip was visually detected inside the chamber based on its color and simultaneously video-recorded with the camera. The jet speed was calculated by measuring the horizontal distance traveled by the colored fluid during the injection. After releasing the foot pedal, the recording was continued until the flow from the tip of the cannula had completely stopped. Experiments were performed using injection pressures ranging from 6 to 20 psi in 2 psi increments. For the baseline condition (BC), each pressure level was tested in triplicate.

Four experimental conditions were investigated:

(1) Baseline condition (BC): ambient pressure in the chamber set at 15 mmHg, “load-and-lock” methodology, and use of the standard viscous fluid injection (VFI) tubing (BL7600 Universal Viscous Fluid Control Pack, Bausch and Lomb, Vaughan, Canada).
(2) Modified parameter 1 (MP1): chamber at ambient pressure and at 45 mmHg.
(3) Modified parameter 2 (MP2): reduction of the VFI tubing connector length to ¼ of the initial length.
(4) Modified parameter 3 (MP3): “lock-and-load” syringe priming methodology.

For the modified parameter configurations (MP1, 2 & 3), experiments were conducted at selected injection pressures of 8, 14, and 20 psi, with four repeat measurements performed at each pressure level.

### Data analysis

All video recordings were visually observed and reviewed for (1) initial flow, (2) jet speed, (3) duration of residual flow after release of the foot pedal, and (4) ambient pressure recording in the injection chamber. Statistical analyses were performed using the GraphPad Prism 10.1.0 for Macintosh software (GraphPad Software, San Diego, CA, USA). One-way analysis of variance (ANOVA) was applied to compare the MP and BC groups.

## Results

### Theoretical modelling results

Using the theoretical model (TM) described in the Methods section, we calculated the hydrodynamic resistance *R*_*hyd*_ of each component in the injection system. The effective resistance *R*_*hyd,eff*_ in the system is the sum of the resistances of each component (Table 1). Using the subretinal injection cannula with a 41g inner diameter polyamide tip (5 mm in length; Model 3219 Polytip Cannula,MedOne Surgical Inc., Sarasota, FL, USA), the resistance equates to 8.43 x10^12^ Pa.s/m^3^*.* Therefore, we identified that 95% of the resistance was located at the tip of the injection cannula. The viscous fluid injection (VFI) tubing (BL7600 Universal Viscous Fluid Control Pack, Bausch and Lomb, Vaughan, Canada) had a negligible effect on hydrodynamic resistance due to its significantly larger diameter. It was connected to the vitrectomy machine (BL15455 Stellaris ELITE, Bausch and Lomb, Vaughan, Canada) and was not considered in any subsequent calculation.

To calculate the flow rate, *Q*, we first needed to establish *P*_min_ (as indicated in equation 3). Using the 1mL syringe (Model 3275 Microdose Injector, MedOne Surgical Inc., Sarasota, FL, USA), we determined that *P*_min_ = 5 + /-1 psi. We also assumed that the friction coefficient was comparable to the static friction coefficient. Thus, we could calculate the flow rate and mean velocity of the fluid exiting the injection system using equations (2) and (4). For the standard setup, the flow rate using water was 0,82 ml/s and the velocity from the cannula tip was 21 cm/s for an injection pressure of 6 psi.

Next, we analyzed how varying the diameter and length of the cannula affected flow at predetermined injection pressures. According to the Hagen-Poiseuille law, reducing the inner diameter *D* to one-half of the initial diameter decreases the flow by a factor of 16. Reducing the cannula tip length to half of the initial length leads to a twofold increase in flow. Table 2 and S1 Table illustrate the effects of these variations on three different types of subretinal injection cannulas used in clinical practice.

**Table 2:** Flow rate *Q* (µl/sec) of a water solution (1mPa.s) at different injection pressure settings using different commercially available subretinal injection cannulas and cannula lengths in baseline conditions (BC).

	6 psi	8 psi	10 psi	12 psi	14 psi	16 psi	18 psi	20 psi
**Subretinal injection cannula with 5 mm length/ 41g polyamide tip (Model 3219, MedOne Surgical Inc.)**	0.82	2.45	4.09	5.73	7.36	9.00	10.64	12.27
**Subretinal injection cannula with 2 mm length/ 41g polyamide tip (Model 3255, MedOne Surgical Inc.)**	1.91	5.72	9.53	13.34	17.16	20.97	24.78	28.60
**Subretinal injection cannula with 0.6 mm length/51g metal tip (Model 3263, MedOne Surgical Inc.)**	0.17	0.51	0.86	1.20	1.54	1.89	2.23	2.57

The administrated volume can be calculated as Q*t_inj_, with t_inj_ = 5 s for all tests.

A final analysis was conducted to determine the effect of viscosity on flow rates, as these vary in gene therapy or cell suspensions used for subretinal delivery. As can be surmised from equation 2, increasing the viscosity to 2, 3, or 4mPa· s resulted in a reduction of flow rates by a factor of 2, 3, or 4, respectively. Saline-based solutions (1 mPa· s) used in gene therapy have a viscosity close to 1mPa· s, whereas 10% dimethyl sulfoxide, often used in cell therapy preparations, has a viscosity close to 2mPa· s*.*

### Experimental model results

**Fig 2:**
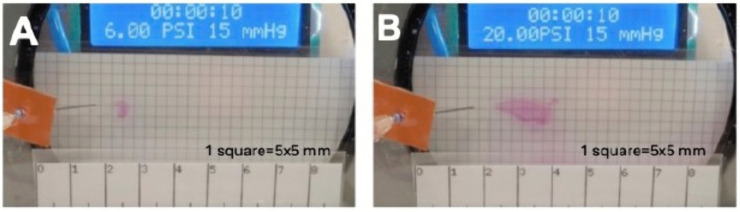
Photographic capture of the flow in the injection chamber at injection pressure levels of 6 psi (A) and 20 psi (B). A minimum injection pressure of 6 psi is required to visually detect flow at the cannula tip within the model **(A)**. Increasing the injection pressure setting on the vitrectomy machine results in a transition to a jet with swirls and eddies **(B)**.

First, we studied the flow exiting from the cannula inside the injection chamber as we increased the injection pressure from 0 to 20 psi in 2 psi increments. Once a minimal pressure of 6 psi was applied, a flow was visually detected at the cannula tip (Fig 2A).

**Fig 3:**
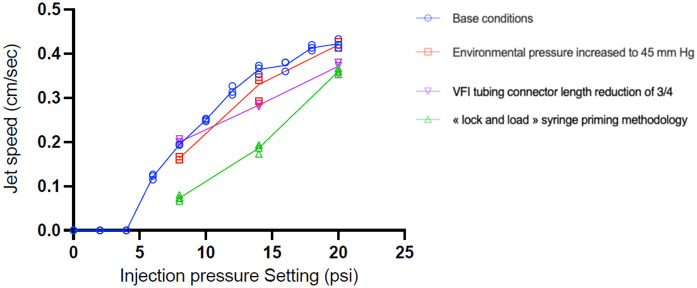
Influence of modified parameters (MP) compared to base conditions (BC) on jet speed (cm/sec). Experimental measurements of jet speed using the model with different injection pressure are shown under base conditions (ambient pressure at 15 mm Hg, standard VFI tubing, and “load-and-lock” priming methodology) and after modifying these parameters: chamber pressure increased from 15 to 45 mm Hg, VFI tubing connector length reduced by three-quarters, and “lock-and-load” syringe priming methodology instead of “load-and-lock”. The error bars are not shown because they are smaller than the size of the data points.

**Fig 4:**
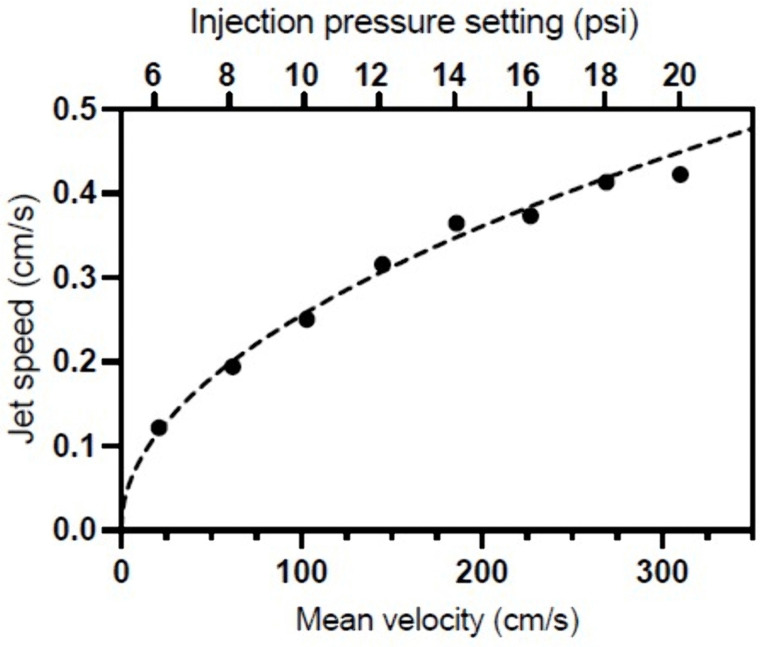
Comparison between the jet speed in the experimental model versus mean velocity in theoretical model. As predicted for laminar flow, the experimental model clearly follows the theoretical prediction of a square-root relationship between the jet speed and the injection pressure. The dashed line corresponds to a value of K = 45.8 for an injection time $t_{inj}$ = 5 sec.

As shown in Figs 3 and 4, the jet speed varied as the square root of the injection pressure under base conditions (BC). Expression (6) was fitted to the experimental points with K=45.8, confirming that with each incremental increase in injection pressure, the increase in jet speed was less pronounced. As the injection pressure increases, the distance traveled by the jet decreases due to the presence of swirls and eddies mainly present in all the experiments above 14 psi, as shown in Fig 2B.

Next, we observed the effects of modifying the baseline parameters on jet speed. Increasing the pressure inside the injection chamber from 15 to 45 mmHg reduced the jet speed at all injection pressures (Fig 3 and S2 Table). At 14 psi, the jet speed was decreased by 0.03 cm/sec (an 8% drop), while at an injection pressure of 8 psi, the reduction was 0.04 cm/sec (a 20% drop). Increasing the pressure inside the injection chamber by 30 mm Hg corresponds to increasing *P*_am_ by 0.58 psi in equation (2). This explains that the decrease in the jet speed is due to the decrease in the flow rate. In other words, it is as if the effective injection pressure decreased by approximately 0.6 psi. This is compatible with the shift observed in Fig 3 between the blue and red curves.

Reducing the length of the VFI tubing to a quarter of its original length did not affect the jet speed at an injection pressure of 8 psi. At higher injection pressures (14 and 20 psi), the jet speed was lower than observed with BC.

In all experimental setups, a residual flow was observed at the cannula tip after releasing the foot pedal from the vitrectomy machine. In BC, as shown in Table 3, the mean time ranged from 27.6 seconds (s) (range: 27–28 s) at an injection pressure of 6 psi to 47.3 s (range 47–48 s) at 20 psi.

**Table 3:** Residual flow duration (seconds + /- sd) after injection at different injection pressure settings (psi) using the Polytip Cannula Model 3219 from MedOne Surgical Inc.

	Base Conditions (BC)	Environmental pressure at 45 mm Hg (MP1)	VFI length reduction of ¾ (MP2)	“Lock-and-load” (MP3)
6 psi	**27.7 + /- 0.47**	–	**–**	**–**
8 psi	**34.7 + /-0.5**	**35.5 + /-1.1**	**39.7 + /-1**	**58.5 + /-0.5**
10 psi	**39 + /- 1.41**	–	–	–
12 psi	**42.3 + /- 0.47**	–	–	–
14 psi	45 + /-0.8	41.7 + /-0.7	44.7 + /-0.8	64.3 + /-1.5
16 psi	**43 + /- 0.82**	–	–	–
18 psi	**45.7 + /- 0.47**	–	–	–
20 psi	**47.3 + /-0.5**	**44.5 + /-1.1**	**47.5 + /-1.1**	**74.3 + /-1**

All experiments except the last column employed the load-and-lock methodology.

**Fig 5:**
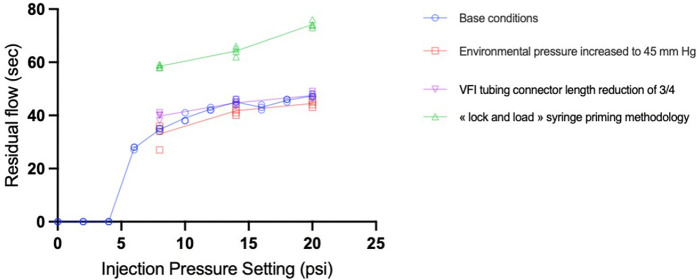
Influence of modified parameters (MP) compared to base conditions (BC) on residual flow(s). Experimental measurements of residual flow using the model with different injection pressure are shown under BC and after modifying these parameters: chamber pressure increased from 15 to 45 mm Hg, VFI tubing connector length was reduced by ¾, and “lock-and-load” syringe priming methodology was applied instead of “load-and-lock”. The error bars are not shown because they are smaller than the size of the data points.

The relationship over the full range of measurements was nonlinear and the best fit was obtained using a logarithmic equation (Fig 5).

The syringe-loading technique substantially affected the residual flow from the cannula tip. Switching from a “load-and-lock” to a “lock-and-load” protocol resulted in an increase in residual flow at all injection pressures (p < 0.0001). As shown in Fig 5, residual flow increased from 50% to 75%, and the difference increased with injection pressure. Table 3 lists the residual flow durations at various injection pressures under different experimental conditions. The loading protocol also significantly affected the injection speed. With the “lock-and-load” priming methodology, flow speed decreased at all measured injection pressures with a larger effect observed at lower injection pressures (p < 0.0001) (Fig 3).

## Discussion

To date, no study has characterized the flow characteristics of subretinal injections. No study has quantified residual flow emanating from the cannula tip after stopping the injection. Clinicians often refer to “reflux” at the end of a subretinal injection, while referring to visible leakage of the solution into the vitreous cavity. This residual flow was to date an unrecognized contributor. The pressure applied to the syringe plunger, the fluid’s viscosity, and the tubing assembly’s exact configuration all influence these parameters. The current study relies on both a theoretical and experimental model to generate data. These models confirm the existence of swirly flow and quantify the presence of residual flow after stopping the injection. The duration of the residual flow depends on the loading strategy.

The theoretical model confirms that 95% of the resistance is concentrated in the distal 41g section of the SI cannula. The length of the cannula tip is directly proportional to resistance and, thus, flow. Doubling the length of the cannula tip reduces flow by half if the flow is laminar, and even more if the flow is turbulent. This underscores the importance of an experimental setup that can semi-quantitatively infer the limitations of the theoretical model and suggest the boundaries to respect when performing subretinal injections. Low injection rate without jetting has been suggested as a crucial aspect to avoid potential retinal damage following subretinal gene therapy [1].

Our study examined injection pressures ranging from 0 to 20 psi, which covers the entire range of clinically relevant values documented in the literature. In 2024, L’Abbate et al. [23] reviewed the mechanical variables that influence subretinal injections. In mouse models, these authors found that a typical injection pressure of 500 hPa (approximately 7.25 psi) is generally effective, with effective pressures ranging from 400 to 1,000 hPa (5.8 to 14.5 psi) [23]. Scruggs et al. reported values of 8 psi for creating a pre-bleb and 5 psi for propagating it in humans [12]. These values fall within the range we validated in our study.

The experimental setup revealed that a minimum pressure of 6 psi is required to initiate fluid flow inside the model. At this pressure, the measured jet speed was 0.12 cm/sec. Previously, Takahashi et al. investigated the effect of subretinal injection pressure on retinal microstructure in a monkey model and reported that a minimum pressure of 6 psi was required for subretinal injections [24]. These authors commented that normal retinal structures were observed on both OCT and histology, at this injection pressure. Conversely, photoreceptor outer segment and retinal pigment epithelium damage were reported with an injection pressure of 20 psi or more. It appears that maintaining a low injection pressure to ensure a controlled, low flow rate in the subretinal space is paramount for atraumatic subretinal injections.

In our experimental system, we observed that a square-root relationship was observed between the jet speed and injection pressure between 6 and 20 psi, which is in accordance with the theoretical prediction (5). This relationship is represented by K=45.8 and $t_{inj}=\text{5}sec,$ as shown in Fig 4.

Olufsen et al. suggested that turbulent flow is likely to be greater than or equivalent to 32 psi [11]. These authors also surmised that turbulence was responsible for the tissue damage observed in the subretinal space of their porcine model, and that stress from fluid flow in the subretinal space adjacent to the RPE is likely an important contributor to this damage. Scruggs et al. reported that the minimum pressure required to initiate a bleb was greater than that required to propagate it [12]. Shear forces can be divided into two components: (1) bleb initiation and (2) subsequent propagation. The shear tolerance of RPE cells has not been studied yet. However, RPE cells tolerated exposure to infusion rates of 5µl/min and shear stress of 6mPa in a microfluidic model [25]. Although the effects of shear stress on RPE cells have not been quantified, only few changes in gene expression were observed in corneal epithelial cells when exposed to 800mPa [26]. If RPE cells behave similarly, they may tolerate the flow rates generated during the bleb expansion phase, which would provide an acceptable safety margin. However, it is unlikely that they can withstand the higher shear stress associated with bleb initiation through a 41g cannula at elevated injection pressures, especially with the cannula tip at the immediate proximity of the RPE. Further research is needed to better understand the RPE cells’ exact tolerance to these stressors [27].

Unintended residual flow was observed in all the experimental setups. The duration of the residual flow depended on the pressure. Injections at 20 psi persisted twice as long as those at 6 psi. Previously, we have demonstrated that withdrawing the cannula from the subretinal space can lead to fluid egress based on the Venturi effect [13]. Here, we have demonstrated that rapid withdrawal causes leakage from a persistent flow. This flow at the cannula tip is caused by the the release of accumulated pressure in the tubing connected to the piston in the syringe. Reflux should ideally be avoided because it allows viral particles to accumulate in the vitreous cavity, which can contribute to an immune response [14–18]. Table 3 helps surgeons estimate the duration of this residual flow based on the chosen injection parameters. A stationary pause can then be included at the end of a subretinal injection prior to retraction of the needle in order to avoid reflux. Depending on the selected injection technique and pressure, the pause could last between 30 s and 75 s.

The syringe priming methodology significantly affects the duration of residual flow. We found that the “lock-and-load” priming method described by Fisher et al. [28] was associated with both a significant decrease in the jet speed and an increase in unintended residual flow as compared to the “load-and-lock” priming methodology. This can be attributed to the presence of small air bubbles, which are invariably present in the syringe when filled in this manner. This can be equated with the concept that the compliance effect is proportional to the volume of trapped air [29]. It is “compressed” when the syringe plunger is pushed, and subsequently regains its original volume, when the plunger is at rest. Therefore, steady-state flow takes longer to reach and persists longer after injection stops.

Intraocular pressure (the ambient pressure in our model) has been reported to have little effect on flow [28]. In our model, careful measurements showed that higher environmental pressures reduced the flow by 20% when the injection pressure was kept as low as possible. This reduction can be explained by the fact that increasing the intraocular pressure decreases the pressure difference that drives the flow, as inferred from equation (2).

The experimental setup has some limitations. First, our model estimates the speed of a fluid jet averaged over a 5 second period, whereas the jet’s exact speed varies over time. Nevertheless, the average jet speed could be related to the calculated mean flow velocity via a single fitting parameter over the entire range of injection pressures. Second, the minimum injection pressure required to observe an injection in our model was similar [24] or slightly inferior [1,30] to those previously reported in animal or human clinical settings. We did not conduct a detailed analysis of the effect of pressure variations within the chamber across the two extreme values of 10 and 45 mmHg, though we did demonstrate that at low flow, pressure variations have an effect. Investigating further the effects of intraocular pressure on flow dynamics may be relevant in a future model, especially considering that IOP can be regulated by the vitrectomy machine itself (“IOP control”), and this parameter can be chosen and optimized independently by the surgeon. Such an approach would also enable a model-based comparison of the respective IOP control performances of different vitrectomy machines.

In our model, there was no inherent retinal adhesive force to counteract the injection. While adequate as a first approximation, retinal adhesivity is an important parameter that requires further characterization but will require a different model system. Retinal adhesive forces may vary significantly between patients, but also in diseased thin retinal or following scaring. The effect of the angle of incidence to the retina could not be analyzed in this model, although this parameter may affect both bleb propagation and retinal tissue damage.

Our experiments were conducted at an ambient temperature of approximately 20°C, whereas the temperature inside the eye is about 37°C. In the context of subretinal injections, fluid injected at room temperature would quickly reach ocular temperature, reducing the viscosity by about 30%. This is also the preferred approach in clinical practice. In 2016, Fischer et al. [28] investigated the influence of temperature on subretinal injections and found that in their optimized injection system, a linear flow rate-to-infusion pressure relationship was maintained across a relevant temperature range (18–36°C).

In our EM, we only tested one type of injection system. There are other commercially available approaches and devices under development that can perform this task. It would be useful to determine the flow characteristics of these alternative devices based on our model. We were indeed unable to obtain precise viscosity data from pharmaceutical companies. Available sources indicate that these solutions, most of which are still under development, likely have a viscosity similar to water. Therefore, we used an aqueous solution in our EM and simulated varying viscosities in our TM.

In conclusion, this study quantitatively characterized the flow dynamics of subretinal injections. Injection pressure significantly impacted both jet velocity and residual flow, the latter of which persisted across all tested pressures. Variations in ambient pressure and syringe priming also affected flow behavior. Minimizing injection pressure and allowing a pause before withdrawing the cannula may reduce procedure-related complications. The length of the pause can be estimated using Table 3. In brief, these results provide a framework for optimizing injection parameters and enhancing the safety of retinal gene therapy applications.

## Supporting information

S1 Table Mean velocity (cm/sec) of a water solution (1mPa/s) at different injection pressure settings using different subretinal injection cannulas. (DOCX)

S2 Table Jet speed (cm/sec + /- sd) after injection at different injection pressure settings (psi) using the Polytip Cannula Model 3219 from MedOne Surgical Inc. (DOCX)
