# Supplementary material for: A theoretical and experimental model of flow characteristics in subretinal injections
Source: PLoS One. 2026 Mar 20;21(3):e0344836. doi: 10.1371/journal.pone.0344836 (PMC13004354; doi:10.1371/journal.pone.0344836)
Supplement: S1 Table — (DOCX) [file pone.0344836.s001.docx]

**S1 Table. Mean velocity (cm/sec) of a water solution (1mPa/s) at different injection pressure settings using different subretinal injection cannulas.**

|  | **6 psi** | **8 psi** | **10 psi** | **12 psi** | **14 psi** | **16 psi** | **18 psi** | **20 psi** |
| --- | --- | --- | --- | --- | --- | --- | --- | --- |
| Subretinal injection cannula with 5mm length/41g polyamide tip (Model 3219, MedOne Surgical Inc.) | 21 | 62 | 103 | 145 | 186 | 227 | 269 | 310 |
| Subretinal injection cannula with 2mm length/41g polyamide tip (Model 3255, MedOne Surgical Inc.) | 48 | 144 | 240 | 337 | 433 | 530 | 626 | 722 |
| Subretinal injection cannula with 0,6 mm length/51g metal tip (Model 3263, MedOne Surgical Inc.) | 28 | 84 | 139 | 195 | 251 | 307 | 362 | 418 |
