# Supplementary material for: A theoretical and experimental model of flow characteristics in subretinal injections
Source: PLoS One. 2026 Mar 20;21(3):e0344836. doi: 10.1371/journal.pone.0344836 (PMC13004354; doi:10.1371/journal.pone.0344836)
Supplement: S2 Table — (DOCX) [file pone.0344836.s002.docx]

**S2 Table. Jet speed (cm/sec +/- sd) after injection at different injection pressure settings (psi) using the Polytip Cannula Model 3219 from MedOne Surgical Inc.**

|  | **Baseline condition (BC)** | **Environmental pressure at 45 mmHg (MP1)** | **VFI length reduction of ¾ (MP2)** | **“Lock-and-load” (MP3)** |
| --- | --- | --- | --- | --- |
| **6 psi** | 0.1216+/-0.006 | - | - | - |
| **8 psi** | 0.194+/-0.001 | 0.163+/-0.004 | 0.200+/-0.005 | 0.073+/-0.006 |
| **10 psi** | 0.250+/-0.003 | - | - | - |
| **12 psi** | 0.316+/-0.010 | - | - | - |
| **14 psi** | 0.364+/-0.010 | 0.330+/-0.025 | 0.283+/-0.004 | 0.187+/-0.010 |
| **16 psi** | 0.373+/-0.012 | - | - | - |
| **18 psi** | 0.413+/-0.007 | - | - | - |
| **20 psi** | 0.422+/-0.010 | 0.420+/-0.005 | 0.372+/-0.012 | 0.360+/-0.006 |

All experiments except the last column employed the load-and-lock methodology.
